# A Highly Flexible Supercapacitor Based on MnO_2_/RGO Nanosheets and Bacterial Cellulose-Filled Gel Electrolyte

**DOI:** 10.3390/ma10111251

**Published:** 2017-10-30

**Authors:** Haojie Fei, Nabanita Saha, Natalia Kazantseva, Robert Moucka, Qilin Cheng, Petr Saha

**Affiliations:** 1 Centre of Polymer Systems, Tomas Bata University in Zlin, Tř. T. Bati 5678, 76001 Zlin, Czech Republic; nabanita@cps.utb.cz (N.S.); kazantseva@cps.utb.cz (N.K.); moucka@cps.utb.cz (R.M.); chengql@ecust.edu.cn (Q.C.); saha@utb.cz (P.S.); 2 Key Laboratory for Ultrafine Materials of Ministry of Education, School of Materials Science and Engineering, East China University of Science and Technology, Shanghai 200237, China

**Keywords:** flexible asymmetric supercapacitor, manganese dioxide, two-dimensional material, reduced graphene oxide, bacterial cellulose, gel electrolyte

## Abstract

The flexible supercapacitors (SCs) of the conventional sandwich-type structure have poor flexibility due to the large thickness of the final entire device. Herein, we have fabricated a highly flexible asymmetric SC using manganese dioxide (MnO_2_) and reduced graphene oxide (RGO) nanosheet-piled hydrogel films and a novel bacterial cellulose (BC)-filled polyacrylic acid sodium salt-Na_2_SO_4_ (BC/PAAS-Na_2_SO_4_) neutral gel electrolyte. Apart from being environmentally friendly, this BC/PAAS-Na_2_SO_4_ gel electrolyte has high viscosity and a sticky property, which enables it to combine two electrodes together. Meanwhile, the intertangling of the filled BC in the gel electrolyte hinders the decrease of the viscosity with temperature, and forms a separator to prevent the two electrodes from short-circuiting. Using these materials, the total thickness of the fabricated device does not exceed 120 μm. This SC device demonstrates high flexibility, where bending and even rolling have no obvious effect on the electrochemical performance. In addition, owing to the asymmetric configuration, the cell voltage of this flexible SC has been extended to 1.8 V, and the energy density can reach up to 11.7 Wh kg^−1^ at the power density of 441 W kg^−1^. This SC also exhibits a good cycling stability, with a capacitance retention of 85.5% over 5000 cycles.

## 1. Introduction

The rapid development of flexible and wearable electronics highly demands flexible energy storage devices [1,2,3,4]. Among various energy storage devices, supercapacitors (SCs) have been considered as one of the most promising candidates because of their high power density, fast charge and discharge rate, and extremely long cycle lifetime [5,6]. Moreover, SCs have advantages in environmental friendliness, safety, and costs. For instance, neutral gel electrolytes have superior safety in terms of undesirable flammability and electrolyte leakage compared with organic electrolytes and acidic or alkaline aqueous/gel electrolytes, which are widely used in flexible SCs [7,8,9,10]. This is extremely significant for wearable electronics in the view of their application in human life. However, as these gel electrolytes are modified from aqueous electrolytes by adding highly viscous and dissolvable polymers, they have the same drawback of the narrow potential window as most aqueous electrolytes, which cannot meet the demands of commercial SCs [11,12]. Aqueous/gel electrolytes have benefited from the development of asymmetric configuration for SCs, as their potential window has been greatly extended [13,14,15,16]. Therefore, a flexible asymmetric SC using neutral gel electrolytes is considered to be a green and effective candidate for portable electronics.

The flexibility is another key characteristic of SCs in wearable electronics [17,18,19]. In order to improve this property, the optimization of the structural design of flexible SCs is required [4]. A conventional flexible SC often consists of two flexible electrodes with current collectors separated by a gel electrolyte. Since this classical sandwich-type flexible SC is constructed by piling up these components layer by layer, the large thickness of the final SC device extremely hinders its flexibility [20]. Therefore, to reduce the thickness of each component is essential. Two-dimensional (2D) nanomaterials such as reduced graphene oxide (RGO) [21], MnO_2_ nanosheets [22], 2D carbides and nitrides (MXenes) [23] and layered vanadyl phosphate (VOPO_4_) [24] become the best choice due to their great ability to form thin films through layer-by-layer stacking, which results in high flexibility and good mechanical properties [18]. Among them, MnO_2_ nanosheets are a low-cost, environmentally friendly and highly attractive positive electrode material for flexible asymmetric SCs with neutral aqueous/gel electrolytes [6,11,25]. A MnO_2_ nanosheet has a layered structure consisting of edge-shared MnO_6_ octahedral layers, guest cation, and bound water, which can facilitate cation intercalation/deintercalation with little structural rearrangement and exhibits a much higher specific capacitance than those of γ-MnO_2_ and β-MnO_2_ [6,25,26,27,28,29,30]. However, as the conductivity of MnO_2_ is low [18,31], achieving a high specific capacitance requires a good distribution of MnO_2_ nanosheets on highly conductive materials (for example Au/cellulose paper [32]) with a large specific surface area. RGO, another 2D nanomaterial with a large surface area and high conductivity, is an excellent choice for constructing such an electronically conductive scaffold for MnO_2_ nanosheets to anchor to [33,34,35,36]. Moreover, a large RGO can be obtained by optimizing the preparation of graphene oxide, which can result in the improved flexibility and enhanced mechanical strength of obtained MnO_2_/RGO composite films [37,38,39].

Although the layer-by-layer tight stacking of 2D nanomaterials highly increases the flexibility and mechanical property of the obtained films, it could significantly hinder the diffusion of electrolyte ions. The study of RGO-based hydrogels proposes a solution for this issue by the reduction of stacking through their separation by water molecules [40,41,42]. The flexible RGO-based hydrogel electrodes can be obtained by compressing wet filtration cakes collected through the vacuum filtration of RGO colloidal suspension [35,42]. Stable RGO colloidal suspensions with various nanoparticles, including MnO_2_, have been successfully prepared by electrostatic repulsion according to previous works, which indicate the promising preparation of a flexible MnO_2_/RGO hydrogel electrode as the positive electrode for asymmetric SCs [22,35,43,44].

In addition, the gel electrolyte layer offers the most space to reduce the thickness without worsening the electrochemical performance of the device. However, when the thickness of the gel electrolyte is reduced, the flexible electrodes are at a high risk of coming into contact with each other during bending, thus creating a short circuit. This is especially an issue with high fluidity gel electrolytes based on non-crosslinked polymers such as polyvinyl alcohol (PVA), polyacrylic acid (PAA), and sodium carboxymethyl cellulose (CMC). Filling gel electrolytes with fibers to form a separator of intertwined fibers is a promising approach to tackle this issue.

In the present study, a flexible asymmetric SC device has been assembled using MnO_2_/RGO (positive electrode) and RGO hydrogel films (negative electrode). A novel polyacrylic acid sodium salt-Na_2_SO_4_ gel electrolyte filled with bacterial cellulose (BC/PAAS-Na_2_SO_4_) was used to reduce the thickness of the electrolyte layer, since the BC network filled in the gel can prevent the contact of the two electrodes during the compression. The assembled flexible device exhibits high flexibility in its sandwich-type construction, benefiting from the thin gel electrolyte layer as well as the use of flexible electrodes piled with 2D nanomaterials. It also displays good electrochemical performance due to its asymmetric configuration and high ionic diffusion in hydrogel electrodes. This device is environmentally friendly, safe, and low-cost due to the appropriate selection of electrode materials and the electrolyte.

## 2. Experimental

### 2.1. Preparation of the Colloidal Suspensions of MnO_2_, RGO and Their Mixture

The colloidal suspension of MnO_2_ nanosheets was prepared through the method reported by K. Kai et al. [45]. Typical procedure was as follows: 12 mL of 1 M tetramethylammonium hydroxide (TMAOH) and 2 mL of 30 wt % H_2_O_2_ were mixed and diluted to 40 mL by deionized water. This mixed solution was then poured to 10 mL of 0.3 M Mn(NO_3_)_2_ under vigorous stirring and kept stirred for 12 h at room temperature. The resulting suspension was dialyzed in deionized water during 3 days with the water periodically changed. Finally, the MnO_2_ colloidal suspension was obtained through separating the precipitate in a centrifuge at 600 rpm (Rotina 380, Hettich, Tuttlingen, Germany). The colloidal suspension of RGO was obtained by directly reducing the graphene oxide in the ammonium solution with hydrazine [46]. Graphene oxide (GO) was prepared from natural graphite flakes (325 meshes, Graphite Týn, Týn nad Vltavou, Czech Republic) by a modified Hummers method [47]. Small-size GO was removed by centrifugation, which was accompanied with pH adjustment [39]. A MnO_2_/RGO mixed colloidal suspension was prepared by mixing these two pure colloids. The mass ratio of the two nanomaterials was 50/50.

### 2.2. Preparation of the Flexible RGO and MnO_2_/RGO Hydrogel Film Electrodes

RGO and MnO_2_/RGO hydrogels were prepared by the vacuum filtration of pure RGO and mixed MnO_2_/RGO colloidal suspensions, respectively. A graphite current collector was deposited on these hydrogels by the successive filtration of graphite flakes suspension well fragmented by ultrasonication. The desired flexible hydrogel electrodes were obtained by compressing these resultant hydrogels between two pieces of polyvinylidene fluoride (PVDF) filter membranes under 15 MPa of pressure.

### 2.3. Preparation of the BC/PAAS-Na_2_SO_4_ Gel Electrolyte

PAAS gel was synthesized by the radical polymerization of acrylic acid in water. Typical procedure was as follows: 3.5 g of acrylic acid was neutralized by NaOH in 8 mL of deionized water. Then, 1.95 g of potassium persulfate (K_2_S_2_O_8_) was added into this solution. The polymerization was conducted at 85 °C with stirring in an N_2_ atmosphere to gain a PAAS gel. Subsequently, 20 g of wet BC membrane was grounded into BC microparticles and precipitated by a centrifugation at 8000 rpm (Rotina 380, Hettich, Tuttlingen, Germany). The BC precipitate and 3.98 g of Na_2_SO_4_ was mixed with the previous PAAS gel electrolyte to obtain a BC/PAAS-Na_2_SO_4_ gel electrolyte.

### 2.4. Fabrication of the Flexible SC Devices

Compressed RGO and MnO_2_/RGO hydrogel films were cut to a rectangle of 3 × 1 (cm) with a rectangular tail of 1.25 × 0.3 (cm), which is for connection to the titanium foils in later measurements. Each piece of RGO and MnO_2_/RGO hydrogel film was placed on a piece of polyethylene (PE) film (40 μm thickness), which lies on a flat plate. The BC/PAAS-Na_2_SO_4_ gel electrolyte was then slightly smeared on the top of hydrogel films. Finally, two flat plates were compressed face-to-face under 0.5 MPa of pressure to obtain a flexible SC device. In this way, the RGO and MnO_2_/RGO hydrogel films were assembled with a traditional sandwich-type structure, separated by a gel electrolyte.

### 2.5. Characterization

The morphologically structural properties of obtained nanomaterials and their composite films were investigated by atomic force microscopy (AFM, Dimension Icon, Bruker, Karlsruhe, Germany), scanning electron microscopy (SEM, FEI Nova NanoSEM450), transmission electron microscopy (TEM, JEOL JEM-2100) and X-ray diffraction (XRD, Rigaku MiniFlex 600). Zeta potentials (Z) of MnO_2_ and RGO in the colloidal suspensions were measured by Zetasizer Nano ZS90 (Malvern, Malvern, UK). The rheological behavior of the BC/PAAS-Na_2_SO_4_ gel electrolyte was examined by a rotational Rheometer (MCR 502, Anton-Paar, Graz, Austria).

The electrochemical characterization was carried out by cyclic voltammetry (CV), a galvanostatic charge–discharge test, and electrochemical impedance spectroscopy (EIS) using Autolab PGSTAT128N (Metrohm, Herisau, Switzerland). The electrochemical performance of the prepared flexible films was firstly investigated in a three-electrode system with an Ag/AgCl reference electrode and a platinum counter electrode in 1 M Na_2_SO_4_. The specific capacitance of the electrodes was calculated from the CV profile using the following equation:

(1) $$C_{sp}=\frac{\int IdU}{2vm\Delta U}$$

where *I* is the current, ∫IdU is the area of the CV curve, *v* is the scan rate, *m* is the mass of the active material (RGO and MnO_2_/RGO), ∆*U* is the potential window, and the factor 2 corrects for the area including both the positive and negative scan. The characterization of assembled devices was carried out in a two-electrode system. The specific capacitance of each device was calculated from the galvanostatic curves at different current densities using the formula:

(2) $$C_{t}=\frac{I\Delta t}{m\Delta V}$$

where *I* is the discharge current, Δ*t* stands for the discharge time, *m* is the total mass of active materials in two electrodes (without graphite current collectors), and Δ*V* is the voltage drop upon discharging (excluding *IR_drop_*, i.e., the potential drop at the beginning of the discharge in charge–discharge profile). The areal capacitance (*C_A_*) of each device was calculated by the following equation: $C_{A}=C_{t}/A$, where *A* is the footprint area of the electrodes. For the symmetric devices, the specific capacitance (*C_sc_*) of one electrode was calculated following the equation: $C_{sc}=4C_{t}$. Finally, the energy density (*E*) and the power density (*P*) of each device were derived from the following equations:

(3) $$E=C_{t}\Delta V^{2}/2$$

(4) P=E/Δt

## 3. Results and Discussion

**Figure 1:**
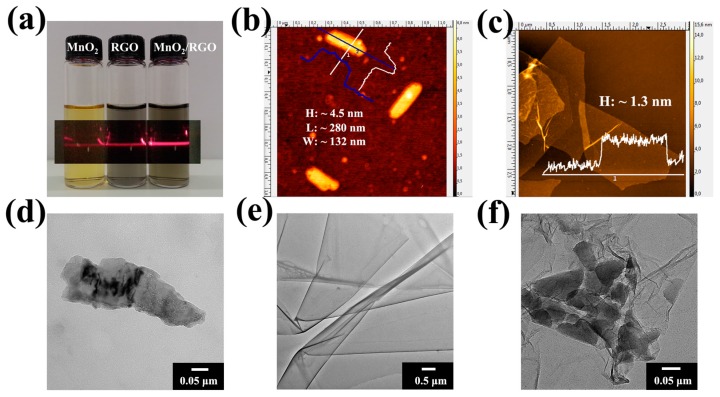
(**a**) Photograph of the aqueous colloidal suspensions of MnO_2_, reduced graphene oxide (RGO), and MnO_2_/RGO, showing the Tyndall effect when the red laser goes through; AFM images of (**b**) MnO_2_ and (**c**) RGO; TEM images of (**d**) MnO_2_, (**e**) RGO and (**f**) MnO_2_/RGO.

In order to prepare RGO and MnO_2_/RGO hydrogel films through the vacuum filtration, firstly, stable RGO and MnO_2_/RGO colloidal suspensions have to be obtained. Figure 1a shows the photograph of RGO and MnO_2_ colloidal suspensions and their mixture (MnO_2_/RGO), respectively. They demonstrate the Tyndall effect when the red laser light goes through the samples, which indicates their colloidal behavior. However, their stability in time is considerably affected by pH. The dependence of the zeta potentials of MnO_2_ and RGO upon pH is present in Figure S1, and their zeta potentials at different pH levels are also summarized in Table S1. The zeta potential of MnO_2_ first deceases (pH = 1.8 to 7.5), and then increases (pH = 7.5 to 11.8). Its lowest zeta potential in the plot is at pH 7.5 (Z = −52.1 mV). Indeed, MnO_2_colloid remains clear without any precipitation at this pH. The stability of MnO colloid decreases at pH from 7.5 to 11.8. It remains stable for about 48 h at pH 11 (−39.4 mV), but only 24 h at pH 11.8 (Z = −35.4 mV). RGO colloid has the opposite behavior in this region (pH from 7.5 to 11.8), and its stability increases with pH (Z = −45.4 mV at pH 11.8, compared with Z = −42.8 mV at pH 11). Therefore, the MnO_2_/RGO mixture was kept at pH 11 and without severely stirring. The lamellar structures of RGO and MnO_2_ were evident from their AFM and TEM images (Figure 1). The height profile scans of AFM images of MnO_2_ and RGO (Figure 1b,c, Figure S2) present a fairly flat surface of both samples, with approximate thicknesses of 4.5 nm and 1.3 nm, respectively. Since the thickness of a single MnO_2_ layer is 0.52 nm [28], the as-prepared MnO_2_ may be formed by several MnO_2_ layers overlapping together, which is also indicated by the TEM image of MnO_2_ from the part of its edge (Figure 1d). Figure 1e displays the transparent, crumpled, and folded structure of RGO, which is typical for RGO. The lateral size of RGO is much larger than MnO_2_. The size distribution of RGO is rather narrow, 2–5 μm after the removal of small-size GO, while it is 100–300 nm of MnO_2_ (Figure S3). The RGO sheets with a large area and flat morphology serve as ideal microscopic substrates to host the MnO_2_ nanosheets. The flat morphology of MnO_2_ and RGO, as well as the electrostatic interaction between them, determines the integration of MnO_2_ nanosheets onto the RGO surface [22]. Indeed, Figure 1f displays that MnO_2_ nanosheets attached to the surface of RGO, rather than aggregated themselves.

**Figure 2:**
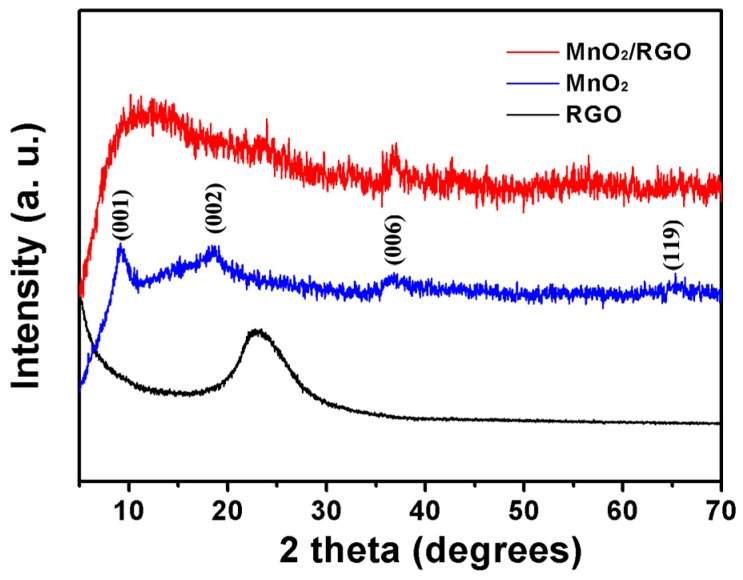
XRD patterns of MnO_2_, RGO, and MnO_2_/RGO.

Figure 2 shows the XRD patterns of RGO, MnO_2_, and MnO_2_/RGO. The XRD pattern of MnO_2_ contains broad peaks at 2θ = 9.2°, 18.4°, 37.0° and 65.4°. These correspond to δ-MnO_2_, which is a 2D layered structure with stabilizing cations intercalated between the MnO_6_ octahedral sheets [27,44]. For the XRD profile of the MnO_2_/RGO sample, a weak peak at 2θ = 23.7° appears, which can be ascribed to the irregular stacking of RGO. More importantly, the (001) and (002) reflection peaks of MnO_2_ decease, and a wide peak appears at the 2θ region of 10–20° [22]. This indicates that the RGO nanosheets restrict the further self-restacking of the MnO_2_ nanosheets, which also implies that the MnO_2_ nanosheets are well integrated into the conductive RGO network.

**Figure 3:**
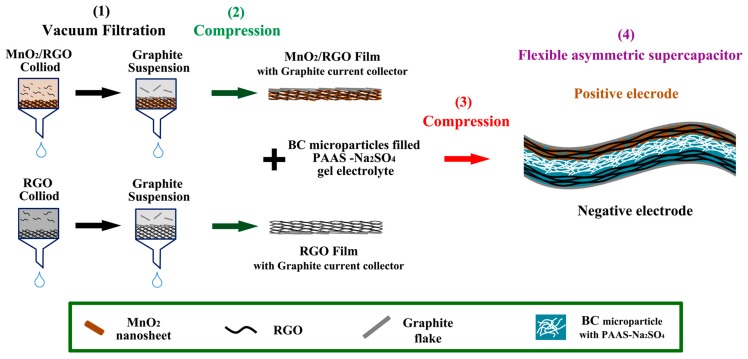
Schematic illustration of the preparation process. (**1**) Vacuum filtration of MnO_2_/RGO, RGO colloidal suspensions and graphite flakes suspension; (**2**) Compression to obtain MnO_2_/RGO and RGO hydrogel films with an attached graphite current collector; (**3**) Compression to assemble the flexible supercapacitor (SC) with obtained hydrogel films and the BC/PAAS-Na_2_SO4 gel electrolyte; (**4**) Schematic structure of the assembled flexible asymmetric SC, demonstrating the role of the separation of bacterial cellulose (BC).

**Figure 4:**
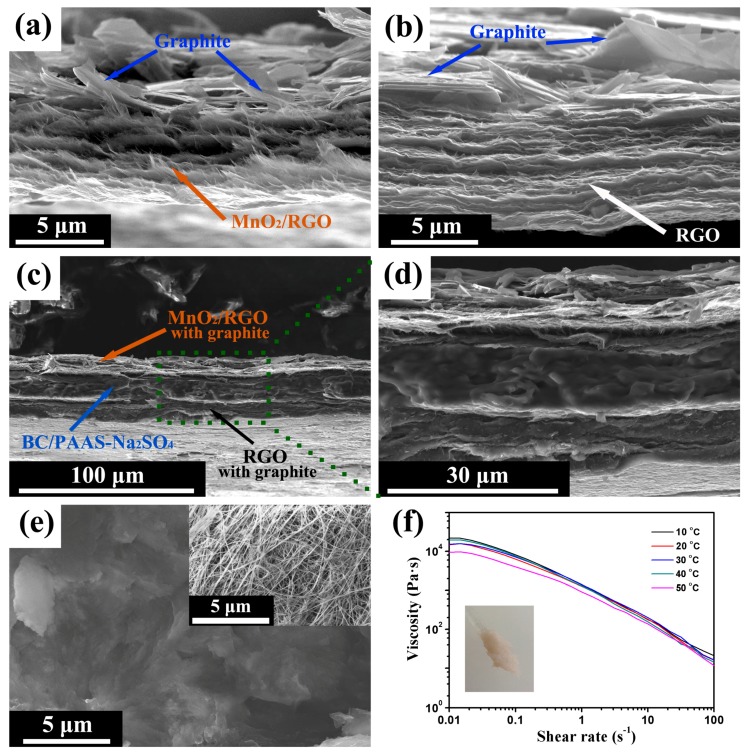
SEM images of the cross-section of MnO_2_/RGO (**a**) and RGO (**b**) hydrogel films with graphite current collectors; and (**c**) the assembled SC and (**d**) at high magnification; and (**e**) the BC/PAAS-Na_2_SO_4_ gel electrolyte (inset image shows the BC nanofibers in BC); (**f**) dependence of the viscosity of BC/PAAS-Na_2_SO_4_ gel electrolyte on shear rate at various temperatures (inset shows the digital image of this gel electrolyte).

Figure 3 demonstrates the fabrication procedure of the flexible asymmetric SC. MnO_2_/RGO (positive electrode) and RGO (negative electrode) hydrogel films are prepared by vacuum filtration of the MnO_2_/RGO and RGO colloidal suspensions, respectively. The graphite current collector is deposited by the successive filtration of graphite flakes suspension, and then well attached to the hydrogel film after the compression, and able to bend with the films. A flexible current collector is one of the key components for flexible supercapacitors [32]. Figure 4a,b show the SEM images of the cross-section of MnO_2_/RGO and RGO films with the graphite current collector on the top surface. They both have a layer-by-layer structure, which benefits from the piling of 2D MnO_2_ and RGO. This also implies that MnO_2_/RGO colloidal suspension stays stable during the filtration without dramatic aggregation. The mass loading of MnO_2_/RGO and RGO is 0.43 mg cm^−2^ and 0.65 mg cm^−2^, respectively. It can be controlled by adjusting the amount of corresponding colloidal suspensions during vacuum filtration, which is very helpful for optimizing the weight ratio of the positive electrodes and negative electrodes in an asymmetric configuration. The cross-section SEM images of the assembled SC (Figure 4c,d) show the sandwich-like structure. The layers of MnO_2_/RGO and RGO, as well as the BC/PAAS-Na_2_SO_4_ gel electrolyte, exhibit uniform thickness at a large scale (Figure 4c). In order to achieve a high flexibility for the final device, the thicknesses of both electrodes, as well as the gel electrolyte, are limited to a few tens of micrometers after the compression (Figure 4d). Importantly, the BC/PAAS-Na_2_SO_4_ gel electrolyte plays a significant role in the assembling of flexible SCs, where two electrodes should be stuck with each other, but also prevented from short circuiting (Figure 3) [48,49]. The BC microparticles soaked in PAAS gel are recognized in Figure 4e, while the inset image shows the BC nanofibers in the pure BC microparticles. The image of BC/PAAS-Na_2_SO_4_ gel electrolyte and its rheological behavior are demonstrated in Figure 4f. It exhibits a very high viscosity (~10^4^ Pa s) and weak temperature dependence. The high viscosity and stickiness of the BC/PAAS-Na_2_SO_4_ gel electrolyte ensure the integration of the two electrodes, which prevents their delamination during the bending cycle. It also enhances the interface between electrode and electrolyte, which is a significant aspect to obtain good electrochemical performance [48,50], but a big challenge to common gel/hydrogel electrolyte membranes. The entanglement of BC nanofibers (Figure 4e (inset)) hinders the decrease of viscosity with temperature caused by PAA polymer chains. It also separates two electrodes when high pressure occurs.

**Figure 5:**
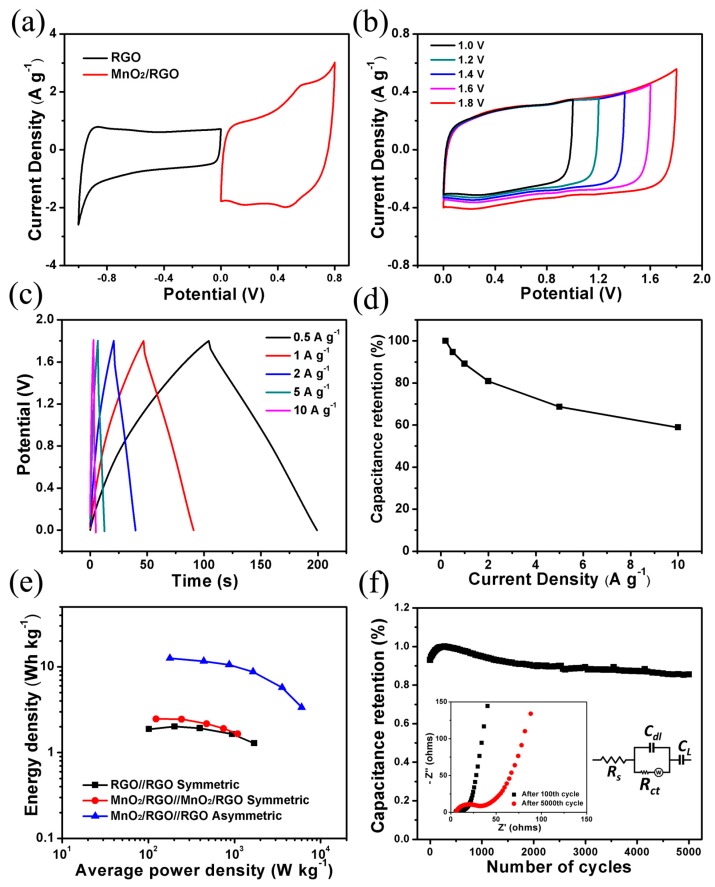
(**a**) Cyclic voltammetry (CV) curves of RGO and MnO_2_/RGO hydrogel films at a scan rate of 10 mV s^−1^ collected in a three-electrode system with an Ag/AgCl reference in 1 M Na_2_SO_4_. Electrochemical performance of the assembled flexible asymmetric SC of MnO_2_/RGO//RGO: (**b**) CV curves at a scan rate of 10 mV s^−1^ with a different potential window; (**c**) Galvanostatic charge–discharge curves at various current densities from 0.5 A g^−1^ to 10 A g^−1^; (**d**) Capacitance retention as a function of discharge currents. (**e**) Ragone plots of the asymmetric device of MnO_2_/RGO//RGO (1.8 V), the symmetric device of RGO//RGO (1 V), and MnO_2_/RGO//MnO_2_/RGO (0.8 V). (**f**) Cycling stability of MnO_2_/RGO//RGO at a current density of 1 A g^−1^ (the inset image shows Nyquist plots before and after 5000 cycles, and the electrical equivalent circuit used for fitting impedance spectra).

The flexible asymmetric SC was fabricated using MnO_2_/RGO and RGO hydrogel films (with graphite current collectors) as the positive and negative electrodes, respectively. First of all, cyclic voltammetry was used to estimate the potential window of each electrode in a three-electrode system, which is shown in Figure 5a. The stable potential window is between −1.0 and 0 V for RGO and between 0 and 0.8 V for MnO_2_/RGO, which indicates that the fabricated device can achieve an extended potential window of 1.8 V. To obtain such operating voltage and keep the amount of charges, *Q*, stored in the positive and negative electrodes, the same is necessary. It can be expressed by the following equation: $Q=C_{sp}{}^{+}m^{+}\Delta U^{+}=C_{sp}{}^{-}m^{-}\Delta U^{-}$, where $\Delta U^{+}$ and $\Delta U^{-}$ represent the potential windows of the positive and negative electrodes, respectively, during the operation of the SC. Thus, the mass ratio of the two electrodes can be calculated by the equation: $m^{-}/m^{+}=C_{sp}{}^{+}\left|\Delta U^{+}\right|/\left(C_{sp}{}^{-}\left|\Delta U^{-}\right|\right)$. Since the $C_{sp}{}^{+}$ of MnO_2_/RGO is 164 F g^−1^ and the $C_{sp}{}^{-}$ of RGO is 87 F g^−1^, which were calculated from the CV profiles in Figure 5a, the weight ratio ($m^{-}/m^{+}$) of RGO and MnO_2_/RGO was kept at 1.5 in the asymmetric SC device, according to the above equation.

As expected, the fabricated asymmetric SC can achieve a wide voltage up to 1.8 V (see Figure 5b). Galvanostatic charge–discharge curves at different current densities in a potential window of 0–1.8 V (Figure 5c) indicate that the assembled asymmetric SC has an excellent capacitive behavior with rapid I–V response. From the slope of a discharge curve, the specific capacitance (*C_t_*) of the asymmetric SC is calculated to be 27 F g^−1^ (*C_A_*, 29 mF cm^−2^), which is based on the total mass of active materials in the two electrodes at a current density of 0.5 A g^−1^, and still reaches 17 F g^−1^ (18 mF cm^−2^) at a high current density of 10 A g^−1^. The specific capacitance as a function of the discharge current of MnO_2_/RGO//RGO asymmetric SCs is summarized in Figure 5d. Ragone plots depicting the relation between power density (*P*) and energy densities (*E*) were used to evaluate the performance of the three types of SCs: RGO//RGO, MnO_2_/RGO//MnO_2_/RGO symmetric, and MnO_2_/RGO//RGO asymmetric SCs, which are shown in Figure 5e. The energy density of MnO_2_/RGO//RGO asymmetric SCs (1.8 V) is much higher than those of RGO//RGO and MnO_2_/RGO//MnO_2_/RGO symmetric SCs (1 V and 0.8 V, respectively). For instance, at a current density of 0.5 A g^−1^, the energy density of MnO_2_/RGO//RGO (11.7 Wh kg^−1^) is about three times higher, compared with that of RGO//RGO (1.9 Wh kg^−1^) and MnO_2_/RGO//MnO_2_/RGO (2.1 Wh kg^−1^). Moreover, MnO_2_/RGO//RGO exhibits a good retention of energy density upon the increase of power density (from 168 W kg^−1^ to 6 kW kg^−1^). The energy density of MnO_2_/RGO//RGO is comparable to those asymmetric SCs based on a general MnO_2_ composite electrode [16,51]. However, it is smaller than those of asymmetric SCs based on electrochemically prepared MnO_2_ [52,53]. The disadvantages of the electrochemical technique are their production limitations and the brittleness of the obtained nanostructured MnO_2_ layers on the flexible substrate upon the increase of deposited film thickness [54,55]. Instead, our assembled SC has an excellent flexibility, which will be discussed afterwards. In addition, MnO_2_/RGO//RGO exhibits excellent cycling stability, which is very important for practical applications. This device shows a good capacitance retention of 85.5% of the maximum capacitance after 5000 cycles. A capacitance increase can be observed in the first 100 cycles, which is ascribed to the cation intercalation/deintercalation in two-dimensional layered MnO_2_ [26,56]. This behavior gives the CV curve of the MnO_2_/RGO a redox pair at about 0.5 V and 0.6 V (Figure 5a and Figure S4) and a higher capacitance. Electrochemical impedance spectroscopy (EIS) was used to investigate the resistance change of MnO_2_/RGO//RGO asymmetric SC before and after the cycling. At high frequencies, the intercept at the real axis (*Z*′) represents the solution resistance (*R_s_*), including the ionic resistance of the electrolyte. The semicircle represents the charge transfer resistance (*R_ct_*) at the electrode–electrolyte interface. Nyquist plots were analyzed by the software ZSimpWin on the basis of an equivalent circuit, which is shown in the inset image in Figure 5f, to obtain the values of *R_s_* and *R_ct_*. After 5000 cycles, nearly no change was observed for *R_s_* (from 5.3 Ω to 5.8 Ω), but an apparent increase of *R_ct_* (from 6.1 to 27.5 Ω) was observed [29,57]. The increase of *R_ct_* is more probably determined by the decrease of contact between the electrode material and the current collector. The increase of *R_ct_* is responsible for the decrease of the energy density of the device.

**Figure 6:**
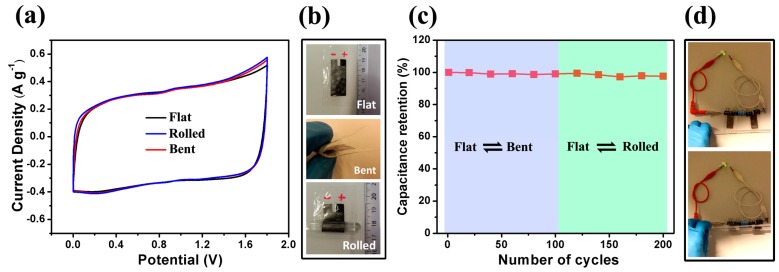
Flexibility of an asymmetric MnO_2_/RGO//RGO device. (**a**) CV curves at 10 mV s^−1^ at three different bending states: (**b**) flat, bent, and rolled; (**c**) Capacitance retention after cycles of repeating flat/bent and flat/rolled; (**d**) Photograph displays a green light-emitting diode (LED) lighted by two asymmetric devices in a series, and demonstrates no obvious performance change of LED from the flat state to rolled state.

For the fabrication of the flexible SC device, PE foils (40 μm) were used as the substrate and encapsulation material. The small thickness and low Young’s modulus of PE foil can reduce the top strain of the flexible electrode on it, and move the mechanical neutral plane close to the interface between the electrode and the substrate [19,58,59,60]. Moreover, due to the small thickness of the final device (about 120 μm including the PE substrates) and the integration (combination and separation) of the two electrodes by BC/PAAS-Na_2_SO_4_, this asymmetric SC exhibits a high flexibility and an excellent cycling stability upon applied deformation (bending and rolling). CV curves have a similar rectangular shape in different deformation states at a scan rate of 10 mV s^−1^ (Figure 6a,b). Moreover, the capacitance of the device after bending and rolling various times does not show significant decrease, which indicates that no substantial damage has taken place during the cycling test (Figure 6c). Figure 6d shows a packing cell with two MnO_2_/RGO//RGO asymmetric SCs in series (3.6 V), which is able to light a light-emitting diode (LED) lamp with a forward voltage of 2.7 V. The rolling of the flexible SC has no obvious effect on the performance of the LED (Video S1).

## 4. Conclusions

A highly flexible asymmetric SC has been fabricated using 2D MnO_2_ and RGO piled hydrogel films and a BC/PAAS-Na_2_SO_4_ neutral gel electrolyte. This SC device demonstrates a high flexibility, where bending and even rolling have no obvious effect on its electrochemical performance. Here, BC/PAAS-Na_2_SO_4_ gel electrolyte plays a significant role in the combination and separation of two electrodes to achieve such flexibility. By asymmetric configuration, the cell voltage of this flexible SC has been extended to 1.8V, and the energy density can reach up to 11.7 Wh kg^−1^, which enhances its potential for practical application. This SC is economical and environmentally friendly due to the use of low-cost MnO_2_ and no harmful neural gel electrolyte. However, in order to obtain high flexibility, the low mass loading of active materials is required, which results in a low areal capacitance (29 mF cm^−2^). Therefore, finding compromise between flexibility and the electrochemical performance of a flexible SC is a goal for our future work.

## Supplementary Materials

The following are available online at www.mdpi.com/1996-1944/10/11/1251/s1. Figure S1: Zeta potential of RGO and MnO_2_ as a function of pH, in aqueous dispersion, adjusted by HCl and NH_3_∙H_2_O. Figure S2: AFM images of MnO_2_ show a uniform thickness of ~4.5 nm; Figure S3: TEM image of MnO_2_ presents a lateral size of 100~300 nm; SEM image of RGO on silicon wafer shows a lateral size of 2~5 μm and their corresponding area distribution. Figure S4: CV curves of MnO_2_/RGO at a scan rate of 10 mV s^−1^ with various cycles collected in a three-electrode system with an Ag/AgCl reference in 1 M Na_2_SO_4_. The redox peaks appear when the potential window extended and increases after cycling. Table S1: Zeta potentials (Z) of RGO and MnO_2_ at various pH, respectively. Video S1 demonstrate that the rolling of the flexible SC has no obvious effect on the performance of the LED.

## References

[1] Jang H., Park Y.J., Chen X., Das T., Kim M.-S., Ahn J.-H. (2016). Graphene-based flexible and stretchable electronics. Adv. Mater..

[2] Lee S.-Y., Choi K.-H., Choi W.-S., Kwon Y.H., Jung H.-R., Shin H.-C., Kim J.Y. (2013). Progress in flexible energy storage and conversion systems, with a focus on cable-type lithium-ion batteries. Energy Environ. Sci..

[3] Zhou G., Li F., Cheng H.-M. (2014). Progress in flexible lithium batteries and future prospects. Energy Environ. Sci..

[4] Wang X., Lu X., Liu B., Chen D., Tong Y., Shen G. (2014). Flexible energy-storage devices: Design consideration and recent progress. Adv. Mater..

[5] Scalia A., Bella F., Lamberti A., Bianco S., Gerbaldi C., Tresso E., Pirri C.F. (2017). A flexible and portable powerpack by solid-state supercapacitor and dye-sensitized solar cell integration. J. Power Sources.

[6] Wang G., Zhang L., Zhang J. (2012). A review of electrode materials for electrochemical supercapacitors. Chem. Soc. Rev..

[7] Zhong C., Deng Y., Hu W., Qiao J., Zhang L., Zhang J. (2015). A review of electrolyte materials and compositions for electrochemical supercapacitors. Chem. Soc. Rev..

[8] Abdallah T., Lemordant D., Claude-Montigny B. (2012). Are room temperature ionic liquids able to improve the safety of supercapacitors organic electrolytes without degrading the performances?. J. Power Sources.

[9] Chang Z., Yang Y., Li M., Wang X., Wu Y. (2014). Green energy storage chemistries based on neutral aqueous electrolytes. J. Mater. Chem. A.

[10] Virya A., Lian K. (2017). Li_2_SO_4_-polyacrylamide polymer electrolytes for 2.0V solid symmetric supercapacitors. Electrochem. Commun..

[11] Dai Z., Peng C., Chae J.H., Ng K.C., Chen G.Z. (2015). Cell voltage versus electrode potential range in aqueous supercapacitors. Sci. Rep..

[12] Gao Q., Demarconnay L., Raymundo-Piñero E., Béguin F. (2012). Exploring the large voltage range of carbon/carbon supercapacitors in aqueous lithium sulfate electrolyte. Energy Environ. Sci..

[13] Long J.W., Bélanger D., Brousse T., Sugimoto W., Sassin M.B., Crosnier O. (2011). Asymmetric electrochemical capacitors—Stretching the limits of aqueous electrolytes. MRS Bull..

[14] Khomenko V., Raymundo-Piñero E., Frackowiak E., Béguin F. (2005). High-voltage asymmetric supercapacitors operating in aqueous electrolyte. Appl. Phys. A.

[15] Yan J., Fan Z., Sun W., Ning G., Wei T., Zhang Q., Zhang R., Zhi L., Wei F. (2012). Advanced Asymmetric Supercapacitors Based on Ni(OH)2/Graphene and Porous Graphene Electrodes with High Energy Density. Adv. Funct. Mater..

[16] Qu Q., Zhang P., Wang B., Chen Y., Tian S., Wu Y., Holze R. (2009). Electrochemical performance of MnO_2_ nanorods in neutral aqueous electrolytes as a cathode for asymmetric supercapacitors. J. Phys. Chem. C.

[17] Niu Z., Zhang L., Liu L., Zhu B., Dong H., Chen X. (2013). All-solid-state flexible ultrathin micro-supercapacitors based on graphene. Adv. Mater..

[18] Peng X., Peng L., Wu C., Xie Y. (2014). Two dimensional nanomaterials for flexible supercapacitors. Chem. Soc. Rev..

[19] Wen L., Li F., Cheng H.-M. (2016). Carbon nanotubes and graphene for flexible electrochemical energy storage: From materials to devices. Adv. Mater..

[20] Zhang H., Qiao Y., Lu Z. (2016). Fully printed ultraflexible supercapacitor supported by a single-textile substrate. ACS Appl. Mater. Interfaces.

[21] Shao Y., El-Kady M.F., Wang L.J., Zhang Q., Li Y., Wang H., Mousavi M.F., Kaner R.B. (2015). Graphene-based materials for flexible supercapacitors. Chem. Soc. Rev..

[22] Peng L., Peng X., Liu B., Wu C., Xie Y., Yu G. (2013). Ultrathin two-dimensional MnO2/graphene hybrid nanostructures for high-performance, flexible planar supercapacitors. Nano Lett..

[23] Zhao M.Q., Ren C.E., Ling Z., Lukatskaya M.R., Zhang C., Van Aken K.L., Barsoum M.W., Gogotsi Y. (2015). Flexible MXene/carbon nanotube composite paper with high volumetric capacitance. Adv. Mater..

[24] Wu C., Lu X., Peng L., Xu K., Peng X., Huang J., Yu G., Xie Y. (2013). Two-dimensional vanadyl phosphate ultrathin nanosheets for high energy density and flexible pseudocapacitors. Nat. Commun..

[25] Wei W., Cui X., Chen W., Ivey D.G. (2011). Manganese oxide-based materials as electrochemical supercapacitor electrodes. Chem. Soc. Rev..

[26] Xiong P., Ma R., Sakai N., Bai X., Li S., Sasaki T. (2017). Redox Active Cation Intercalation/Deintercalation in Two-dimensional layered MnO_2_ nanostructures for high-rate electrochemical energy storage. ACS Appl. Mater. Interfaces.

[27] Devaraj S., Munichandraiah N. (2008). Effect of crystallographic structure of MnO_2_ on its electrochemical capacitance properties. J. Phys. Chem. C.

[28] Omomo Y., Sasaki T., Zhou L., Watanabe M. (2003). Redoxable nanosheet crystallites of MnO_2_ derived via delamination of a layered manganese oxide. J. Am. Chem. Soc..

[29] Athouël L., Moser F., Dugas R., Crosnier O., Bélanger D., Brousse T. (2008). Variation of the MnO_2_ birnessite structure upon charge/discharge in an electrochemical supercapacitor electrode in aqueous Na_2_SO_4_ electrolyte. J. Phys. Chem. C.

[30] Huang M., Zhang Y., Li F., Zhang L., Ruoff R.S., Wen Z., Liu Q. (2014). Self-assembly of mesoporous nanotubes assembled from interwoven ultrathin birnessite-type MnO_2_ nanosheets for asymmetric supercapacitors. Sci. Rep..

[31] Cao J., Li X., Wang Y., Walsh F.C., Ouyang J.-H., Jia D., Zhou Y. (2015). Materials and fabrication of electrode scaffolds for deposition of MnO2 and their true performance in supercapacitors. J. Power Sources.

[32] Ko Y., Kwon M., Bae W.K., Lee B., Lee S.W., Cho J. (2017). Flexible supercapacitor electrodes based on real metal-like cellulose papers. Nat. Commun..

[33] Kuila T., Mishra A.K., Khanra P., Kim N.H., Lee J.H. (2013). Recent advances in the efficient reduction of graphene oxide and its application as energy storage electrode materials. Nanoscale.

[34] Lee J.W., Hall A.S., Kim J.-D., Mallouk T.E. (2012). A Facile and Template-free hydrothermal synthesis of Mn_3_O_4_ nanorods on graphene sheets for supercapacitor electrodes with long cycle stability. Chem. Mater..

[35] Gao H., Xiao F., Ching C.B., Duan H. (2012). Flexible all-solid-state asymmetric supercapacitors based on free-standing carbon nanotube/graphene and Mn_3_O_4_ nanoparticle/graphene paper electrodes. ACS Appl. Mater. Interfaces.

[36] Saravanakumar B., Purushothaman K.K., Muralidharan G. (2016). Fabrication of two-dimensional reduced graphene oxide supported V_2_O_5_ networks and their application in supercapacitors. Mater. Chem. Phys..

[37] Cai M., Thorpe D., Adamson D.H., Schniepp H.C. (2012). Methods of graphite exfoliation. J. Mater. Chem..

[38] Xiang C., Young C.C., Wang X., Yan Z., Hwang C.-C., Cerioti G., Lin J., Kono J., Pasquali M., Tour J.M. (2013). Large flake graphene oxide fibers with unconventional 100% knot efficiency and highly aligned small flake graphene oxide fibers. Adv. Mater..

[39] Wang X., Bai H., Shi G. (2011). Size fractionation of graphene oxide sheets by pH-assisted selective sedimentation. J. Am. Chem. Soc..

[40] Du P., Liu H.C., Yi C., Wang K., Gong X. (2015). Polyaniline-modified oriented graphene hydrogel film as the free-standing electrode for flexible solid-state supercapacitors. ACS Appl. Mater. Interfaces.

[41] Xu Y., Lin Z., Huang X., Liu Y., Huang Y., Duan X. (2013). Flexible solid-state supercapacitors based on three-dimensional graphene hydrogel films. ACS Nano.

[42] Wang Y., Yang X., Qiu L., Li D. (2013). Revisiting the capacitance of polyaniline by using graphene hydrogel films as a substrate: The importance of nano-architecturing. Energy Environ. Sci..

[43] Lee Y.R., Kim I.Y., Kim T.W., Lee J.M., Hwang S.J. (2012). Mixed colloidal suspensions of reduced graphene oxide and layered metal oxide nanosheets: Useful precursors for the porous nanocomposites and hybrid films of graphene/metal oxide. Chem. Eur. J..

[44] Tang Q., Sun M., Yu S., Wang G. (2014). Preparation and supercapacitance performance of manganese oxide nanosheets/graphene/carbon nanotubes ternary composite film. Electrochim. Acta.

[45] Kai K., Yoshida Y., Kageyama H., Saito G., Ishigaki T., Furukawa Y., Kawamata J. (2008). Room-temperature synthesis of manganese oxide monosheets. J. Am. Chem. Soc..

[46] Li D., Muller M.B., Gilje S., Kaner R.B., Wallace G.G. (2008). Processable aqueous dispersions of graphene nanosheets. Nat. Nanotechnol..

[47] Eigler S., Enzelberger-Heim M., Grimm S., Hofmann P., Kroener W., Geworski A., Dotzer C., Rockert M., Xiao J., Papp C. (2013). Wet chemical synthesis of graphene. Adv. Mater..

[48] Gao H., Lian K. (2014). Proton-conducting polymer electrolytes and their applications in solid supercapacitors: A review. RSC Adv..

[49] Lv X., Li G., Li D., Huang F., Liu W., Wei Q. (2017). A new method to prepare no-binder, integral electrodes-separator, asymmetric all-solid-state flexible supercapacitor derived from bacterial cellulose. J. Phys. Chem. Solids.

[50] Sacco A., Bella F., De La Pierre S., Castellino M., Bianco S., Bongiovanni R., Pirri C.F. (2015). Electrodes/Electrolyte Interfaces in the Presence of a Surface-Modified Photopolymer Electrolyte: Application in Dye-Sensitized Solar Cells. ChemPhysChem.

[51] Brousse T., Taberna P.-L., Crosnier O., Dugas R., Guillemet P., Scudeller Y., Zhou Y., Favier F., Bélanger D., Simon P. (2007). Long-term cycling behavior of asymmetric activated carbon/MnO_2_ aqueous electrochemical supercapacitor. J. Power Sources.

[52] Gao H., Xiao F., Ching C.B., Duan H. (2012). High-performance asymmetric supercapacitor based on graphene hydrogel and nanostructured MnO_2_. ACS Appl. Mater. Interfaces.

[53] El-Kady M.F., Ihns M., Li M., Hwang J.Y., Mousavi M.F., Chaney L., Lech A.T., Kaner R.B. (2015). Engineering three-dimensional hybrid supercapacitors and microsupercapacitors for high-performance integrated energy storage. Proc. Natl. Acad. Sci. USA.

[54] Dong L., Xu C., Li Y., Huang Z.-H., Kang F., Yang Q.-H., Zhao X. (2016). Flexible electrodes and supercapacitors for wearable energy storage: A review by category. J. Mater. Chem. A.

[55] Lokhande C.D., Dubal D.P., Joo O.-S. (2011). Metal oxide thin film based supercapacitors. Curr. Appl. Phys..

[56] Hsu Y.K., Chen Y.C., Lin Y.G., Chen L.C., Chen K.H. (2011). Reversible phase transformation of MnO_2_ nanosheets in an electrochemical capacitor investigated by in situ Raman spectroscopy. Chem. Commun..

[57] Wei W., Cui X., Chen W., Ivey D.G. (2009). Electrochemical cyclability mechanism for MnO_2_ electrodes utilized as electrochemical supercapacitors. J. Power Sources.

[58] Koo M., Park K.-I., Lee S.H., Suh M., Jeon D.Y., Choi J.W., Kang K., Lee K.J. (2012). Bendable inorganic thin-film battery for fully flexible electronic systems. Nano Lett..

[59] Suo Z., Ma E.Y., Gleskova H., Wagner S. (1999). Mechanics of rollable and foldable film-on-foil electronics. Appl. Phys. Lett..

[60] Gleskova H., Cheng I.C., Wagner S., Suo Z., Wong W.S., Salleo A. (2009). Mechanical theory of the film-on-substrate-foil structure: Curvature and overlay alignment in amorphous silicon thin-film devices fabricated on free-standing foil substrates. Flexible Electronics: Materials and Applications.

