# Supplementary material for: A Highly Flexible Supercapacitor Based on MnO2/RGO Nanosheets and Bacterial Cellulose-Filled Gel Electrolyte
Source: Materials (Basel). 2017 Oct 30;10(11):1251. doi: 10.3390/ma10111251 (PMC5706198; doi:10.3390/ma10111251)
Supplement: Supplementary file 1 [file materials-10-01251-s001.zip › materials-235315-supplementary/materials-235315-supplementary- proof done - Selina/materials-235315-Supplementary Materials.pdf]

Article

A Highly Flexible Supercapacitor Based on MnO_2/RGO Nanosheets and Bacterial Cellulose-Filled Gel Electrolyte

Haojie Fei [1,*], Nabanita Saha [1], Natalia Kazantseva [1], Robert Moucka [1], Qilin Cheng [1,2] and Petr Saha [1]

1 Centre of Polymer Systems, Tomas Bata University in Zlin, Tř. T. Bati 5678, 76001 Zlin, Czech Republic; nabanita@cps.utb.cz (N.S.); kazantseva@cps.utb.cz (N.K.); moucka@cps.utb.cz (R.M.); chengql@ecust.edu.cn (Q.C.); saha@utb.cz (P.S.)

2 Key Laboratory for Ultrafine Materials of Ministry of Education, School of Materials Science and Engineering, East China University of Science and Technology, Shanghai 200237, China

* Correspondence: haojie@cps.utb.cz; Tel.: +420-57603-8156

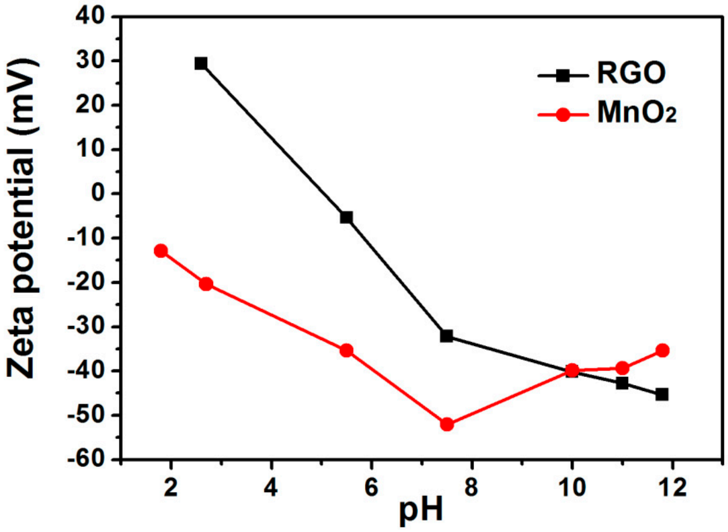

Figure S1. Zeta potential of RGO and MnO_2 as a function of pH, in aqueous dispersion, adjusted by HCl and $NH_3 \cdot H_2O$

Table S1. Zeta potentials (Z) of RGO and MnO_2 at various pH, respectively.

pH	1.8	2.6	2.7	5.5	7.5	10	11	11.8
Z of RGO (mV)	–	29.4	–	-5.4	-32.2	-40.2	-42.8	-45.4
Z of MnO_2 (mV)	-12.9	–	-20.4	-35.4	-52.1	-39.8	-39.4	-35.4

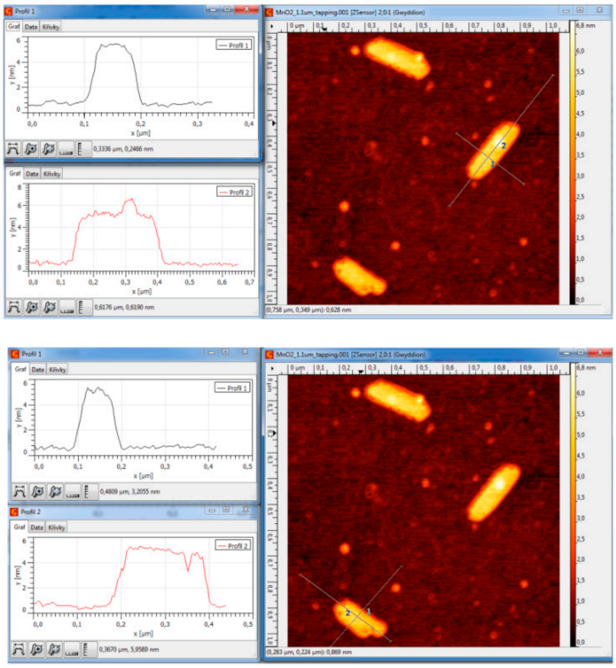

Figure S2. AFM images of MnO_2 show a uniform thickness of ~ 4.5 nm.

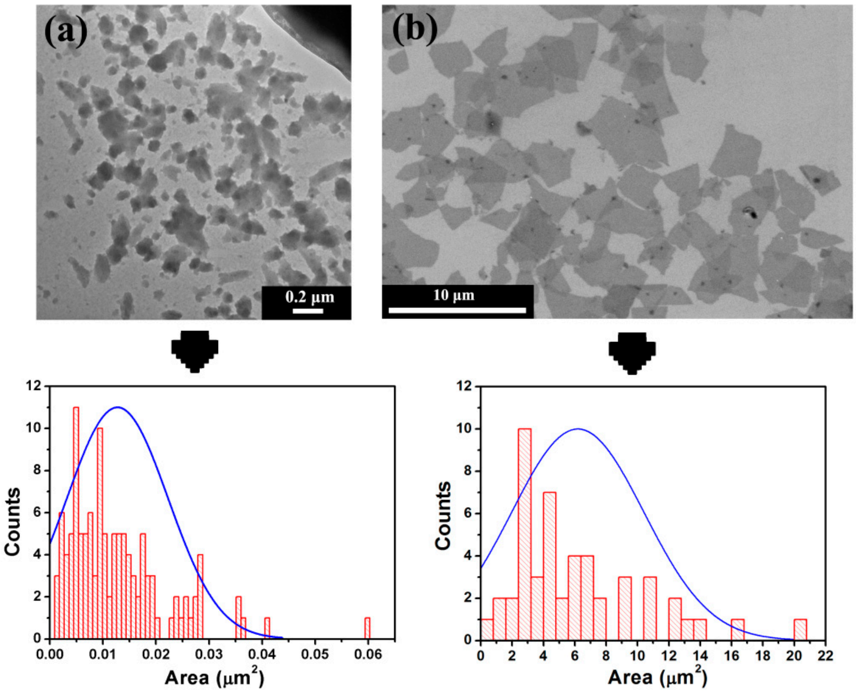

Figure S3. **(a)** TEM image of MnO_2 presents a lateral size of 100 ~ 300 nm; **(b)** SEM image of RGO on silicon wafer shows a lateral size of 2 ~ 5 µm and their corresponding area distribution.

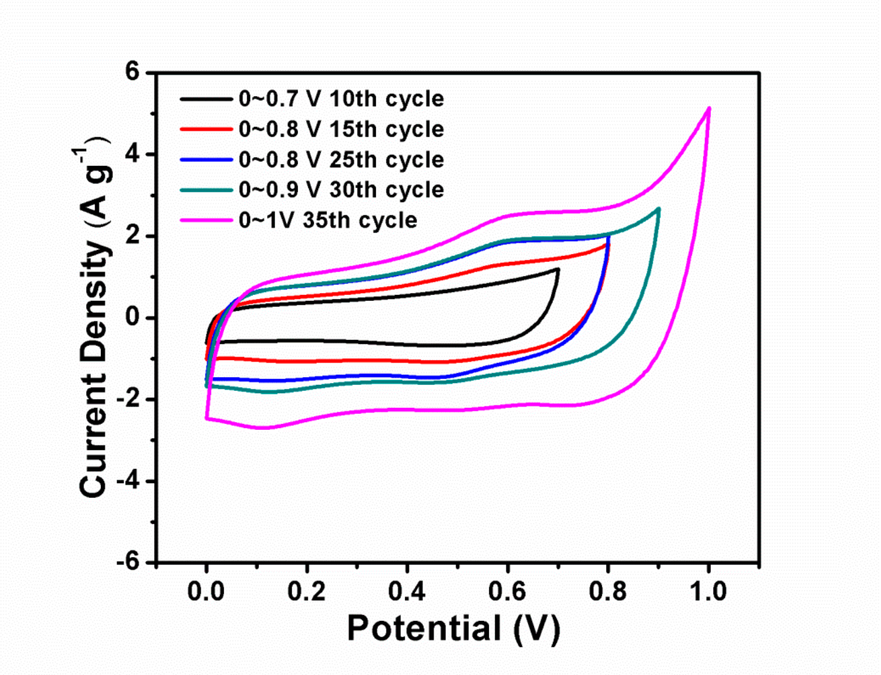

Figure S4. CV curves of MnO_2/RGO at a scan rate of 10 mV s^{-1} with various cycles collected in a three-electrode system with an Ag/AgCl reference in 1 M Na_2SO_4. The redox peaks appear when the potential window extended and increases after cycling.
